# Chemical Reactions Catalyzed by Metalloporphyrin-Based Metal-Organic Frameworks

**DOI:** 10.3390/molecules18067279

**Published:** 2013-06-21

**Authors:** Shirley Nakagaki, Gabriel Kaetan Baio Ferreira, Geani Maria Ucoski, Kelly Aparecida Dias de Freitas Castro

**Affiliations:** Laboratório de Bioinorgânica Grupo de Bioinorgânica e Catálise, Departamento de Química, Universidade Federal do Paraná, CP 19081, CEP 81531-990, Curitiba, PR, Brazil

**Keywords:** porphyrin, heterogeneous catalysis, oxidation, metal-organic framework

## Abstract

The synthetic versatility and the potential application of metalloporphyrins (MP) in different fields have aroused researchers’ interest in studying these complexes, in an attempt to mimic biological systems such as cytochrome P-450. Over the last 40 years, synthetic MPs have been mainly used as catalysts for homogeneous or heterogeneous chemical reactions. To employ them in heterogeneous catalysis, chemists have prepared new MP-based solids by immobilizing MP onto rigid inorganic supports, a strategy that affords hybrid inorganic-organic materials. More recently, materials obtained by supramolecular assembly processes and containing MPs as building blocks have been applied in a variety of areas, like gas storage, photonic devices, separation, molecular sensing, magnets, and heterogeneous catalysis, among others. These coordination polymers, known as metal-organic frameworks (MOFs), contain organic ligands or complexes connected by metal ions or clusters, which give rise to a 1-, 2- or 3-D network. These kinds of materials presents large surface areas, Brønsted or redox sites, and high porosity, all of which are desirable features in catalysts with potential use in heterogeneous phases. Building MOFs based on MP is a good way to obtain solid catalysts that offer the advantages of bioinspired systems and zeolitic materials. In this mini review, we will adopt a historical approach to present the most relevant MP-based MOFs applicable to catalytic reactions such as oxidation, reduction, insertion of functional groups, and exchange of organic functions.

## 1. Metalloporphyrins as Bioinspired Systems

**Figure 1:**
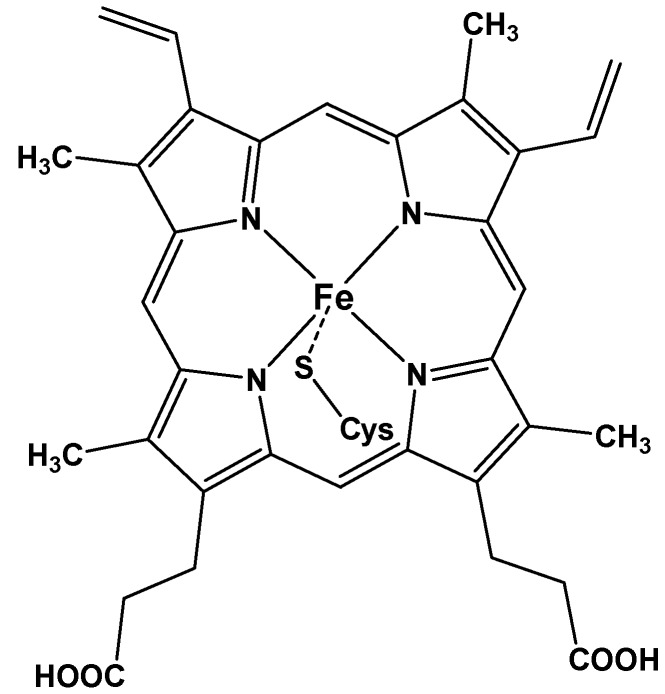
Schematic representation of the prosthetic group of the cysteinate-heme enzyme cytochrome P-450.

Since their discovery in the 1960s, several works have been conducted to isolate and characterize the enzymes belonging to the cytochrome P-450 family [1,2]. This superfamily is constituted by cysteinato-heme enzymes and exists in all forms of life; e.g., plants, bacteria, and mammals. P-450 enzymes play a key role in the oxidative transformation of endogeneous and exogeneous molecules [3]; their active site contains an iron(III) protoporphyrin-IX covalently linked to the protein by the sulfur atom of a proximal cysteine ligand (Figure 1) [3]. These enzymes are able to catalyze several reactions such as monooxygenation (mainly the hydroxylation of saturated carbon-hydrogen bonds, and epoxidation of unsaturated bonds), dehydrogenation, C=N bond cleavage, and oxidative deformylation; they display peroxidase and oxidase activity [4].

Inspired by the reactivity of this biological system, chemists have concentrated efforts on designing routes to obtain new synthetic porphyrins and metalloporphyrins (MPs), aiming to mimic the catalytic activity of cytochrome P-450 [5,6]. Studies on the native enzyme and iron porphyrin model systems have helped scientists understand how cytochrome P-450 enzymes activate dioxygen and oxidize substrates [1,3].

### 1.1. Synthetic Porphyrins

Porphyrins are versatile compounds with potential use in different fields like medicine, catalysis, and electronics. The myriads of applications of these compounds stem from their singular physical and chemical characteristics, such as high stability, intense electronic absorption and emission, rigid planar geometry, and reactivity, among others. Over recent years, numerous processes for the synthesis of porphyrins have been developed.

Porphyrins are highly conjugated tetrapyrrolic macrocycles consisting of four pyrrole rings linked via methine bridges. Alternating single and double bonds confer stability to the porphyrinic ring through resonance structures. A large number of porphyrins with different structures and features can be isolated from Nature [7] or synthesized in the laboratory [8,9,10]. Porphyrins can be obtained via one of two general routes: (i) a reaction involving a pyrrole intermediate [11,12] or (ii) chemical modification of naturally-occurring or synthetic porphyrins [13]. Furthermore, diverse porphyrinic macrocycles can be achieved by electrophilic aromatic substitution (nitration [14], halogenations [15], sulfonation [16], formylation [17], and acylation [18]), nucleophilic addition [19], nucleophilic aromatic substitution [20], and cycloaddition reactions [21].

In the 1970s, Groves *et al.* [22] published the first paper on the use of a synthetic iron porphyrin as catalyst for the epoxidation of alkenes and hydroxylation of alkanes by iodosylbenzene. Since then, many authors have employed MPs with different metals to catalyze the oxidation of various organic substrates. New synthetic routes have also been designed, to improve the catalytic performance of these complexes. 

**Figure 2:**
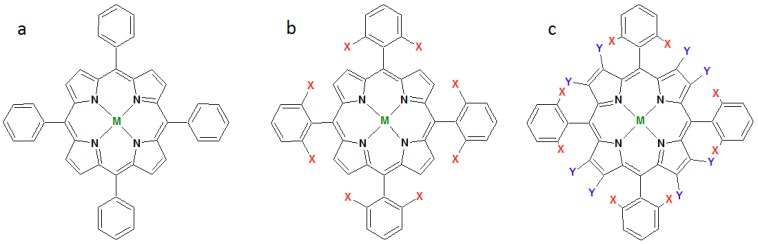
Metalloporphyrins of the (**a**) first, (**b**) second, and (**c**) third generation, where X represents an EWG or bulky group and Y is a halogen atom.

In 1997, Dolphin and Traylor [8] proposed a classification for the many MPs used in catalysis on the basis of their structures. These authors designated the first synthetic MP that Groves [22] employed in cytochrome P-450 biomimetic catalysis, [Fe(TPP)]Cl, as a first-generation porphyrin [Figure 2(a)]. This complex affords modest catalytic results, because the fragile porphyrin structure is easily destroyed under the oxidizing conditions of the catalytic reaction. 

Dolphin and Traylor [8] classified mesophenyl-substituted MP bearing electronegative and/or bulky groups as second-generation porphyrins [Figure 2(b)]. Such complexes afford fantastic catalytic results, mainly in the most difficult of all the oxidation reactions–alkane hydroxylation. Second-generation MPs perform better than first-generation catalysts because: (i) electron-withdrawing groups (EWG); e.g., halogen atoms make the catalytic intermediate species more electrophilic and therefore a more powerful oxidizing species; (ii) the bulky groups at the phenyl substituents avoid intermolecular interactions that can generate inactive catalytic species or promote the auto-oxidative destruction of MP in solution. Together, these two factors confer the second-generation porphyrins a more robust nature [8].

The introduction of electronegative groups in the β-pyrrole positions of the ring of second-generation porphyrins give rise to porphyrin ligands of the third generation [Figure 2(c)]. At first, it was expected that the addition of extra EWG or bulky groups would render the macrocycle ring even more robust and resistant to oxidative self-destruction and increase the catalytic activity of MP, but most of the communications on third-generation MPs published in the past two decades have revealed that they do not furnish the same catalytic results as the second-generation counterparts [23,24,25,26,27,28,29,30,31]. In homogeneous catalysis, third-generation MP undergo inactivation for several reasons, consequently providing poor catalytic yields.

### 1.2. Metalloporphyrins as Catalysts for Oxidation Reactions in Homogenous Phase

The oxidation of alkenes to epoxides is an important process from an economic standpoint: epoxides are useful intermediates when producing high-value commercial polymers like polyurethane, polyamides, epoxy resins, and polyesters. Similarly, the oxidation of highly inert alkanes gives alcohols and ketones, which are essential to obtain the fibers Nylon 6 and Nylon 6.6. In industry, alkane oxidation is usually conducted under high pressure and temperature, which results in low yields of the main products, together with large amounts of byproducts [32,33].

Therefore, attempts have been made to obtain efficient and selective catalysts that can oxidize alkanes under mild conditions, with a view to industrial applications. MPs stand out among the molecules that have been investigated over the last 30 years: in particular, iron and manganese porphyrins selectively catalyze the oxidation of cyclohexane to the corresponding alcohol and/or ketone, under mild conditions. Indeed, MP catalysts do not generate any other byproducts, and alcohol is the main or even the sole product in most cases [26,32,34,35]. Furthermore, immobilizing the MP onto selected rigid and inert inorganic supports can tailor the selectivity of the resulting catalyst, depending on the structure of the matrix [36,37].

Years after cytochrome P-450 was reported to catalyze the monooxygenation of substrates by iodosylbenzene [1,38], Groves [6,22] showed that the first-generation [Fe(TPP)]Cl catalyzes the epoxidation of *cis*-stilbene and the hydroxylation of adamantane by PhIO. Hence, this author was able to (i) mimic the intermediate iron complexes participating in the catalytic cycle of cytochrome P-450, (ii) understand the chemistry of P-450, and (iii) mimic the P-450-dependent oxidation reactions [1].

To obtain efficient biomimetic systems, one has to consider such aspects as the choice of metal and porphyrin ligand. Among the many porphyrin systems developed to date, Fe, Mn, and Ru complexes furnish the best catalytic results regarding oxidation reactions. As for the porphyrinic macrocycle, the synthesis of three generations of porphyrin ligands has provided catalysts that are more resistant to oxidative degradation and that can efficiently oxidize substrates [1].

Choosing the oxidizing agent; *i.e.*, the oxygen atom donor, is another important factor when it comes to the MP-catalyzed oxidation of hydrocarbons. Different compounds have been used as oxygen donors–iodosylarenes, hypochlorites, amine N-oxides, peracids, potassium monopersulfate, alkylhydroperoxides, and H_2_O_2_. Depending on the oxidant employed in the catalytic reaction, it might be necessary to use a co-catalyst. For example, when employing hydroperoxides as oxygen donor, a co-catalyst might be essential to facilitate the heterolytic cleavage of the O-O bond and to transfer one oxygen atom to the metal, to generate the active catalytic species [26,39,40]. 

Metalloporphyrins have also found application to selectively oxidize a wide variety of substrates in fine chemistry, degrade pollutants [41,42,43,44], and cleave DNA [45].

In homogeneous catalytic systems, the catalyst and all the other reactants remain in the same phase; they are expected to interact easily, which should improve the catalytic activity and culminate in highly efficient and selective reactions. However, several factors can limit homogeneous catalysis based on MPs, such as the MP ring structure, MP solubility in the reaction medium, and MP intermolecular interactions–the latter can deactivate the catalyst by self-destruction or form inactive species. Another difficulty inherent to homogeneous systems is MP recovery from the reaction medium after the reaction; in most cases, the precious catalyst is lost. To overcome these problems, one strategy is to synthesize MPs that are more stable and efficient, the so-called second-generation MPs. Another interesting approach to make MP more robust is to immobilize them onto inorganic or organic supports [46,47,48,49,50]. This immobilization offers an extra bonus: the catalyst can be recovered from the reaction medium and reused in many catalytic cycles, paving the way for the technological application of this family of catalysts. 

In the 1980s, Nolte and Drenth [51] published the first study on the epoxidation of alkenes catalyzed by a manganese porphyrin bound to a polyisocyanide polymer in the presence of 4-methyl-pyridine, which is considered the pioneering work regarding the development of supported MPs. Two strategies help minimize the deactivating effects operating in homogeneous catalysis: (1) changing the phase of the catalytic complex through its heterogenization on an inert solid, or (2) processing the catalytic reaction mixtures to generate insoluble solids.

MP heterogenization can be accomplished via chemical or physical processes. Chemical processes generally involve formation of a chemical (electrostatic or covalent) bond between the support and the catalyst; physical processes include adsorption, intercalation, and encapsulation. Compared with physical interactions, an effective chemical interaction is expected to render a more robust and resistant solid catalyst; it should avoid catalyst leaching and allow it to be reused several times [52,53,54,55].

Zeolites [56,57,58], silicas, mineral clays, and lamellar compounds are examples of the interesting catalyst supports used [56,59,60,61,62,63]. 

Materials based on the active catalytic molecules only, such as coordination polymers (CP) and metal-organic frameworks (MOFs), are good ways to obtain solid heterogeneous catalysts and avoid the catalyst dilution typical of immobilized MP [64].

In this mini-review we will present a brief bibliography of the recent efforts put into the development of MOFs based on MPs as building blocks. We will outline some synthetic approaches used to prepare these MOFs; we will also revise their application in catalysis.

## 2. Advances in the Field of Coordination Polymers and Metal-Organic Frameworks

As discussed above, MP can catalyze some kinds of chemical reactions. Currently, there is an increasing need for robust and recyclable non-diluted solid systems that can function as catalysts in heterogeneous processes. Such systems can be achieved by using a new class of porphyrin-based materials, namely coordination polymers (CPs), to obtain MOFs having MPs as building blocks.

Let us first introduce some definitions that will help understand the classifications to be used further in this review. According to Corma *et al.*, coordination polymers (CPs) “are solid materials formed by an extended network of metal ions (or clusters) coordinated to multidentate organic molecules” [64]. This definition does not differentiate between crystalline and amorphous materials, porous and nonporous structures, or robust and unstable solids. MOFs, on the other hand, can be defined as a subclass of CPs, “which are crystalline and porous compounds involving strong metal-ligand interactions.” [64,65].

**Figure 3:**
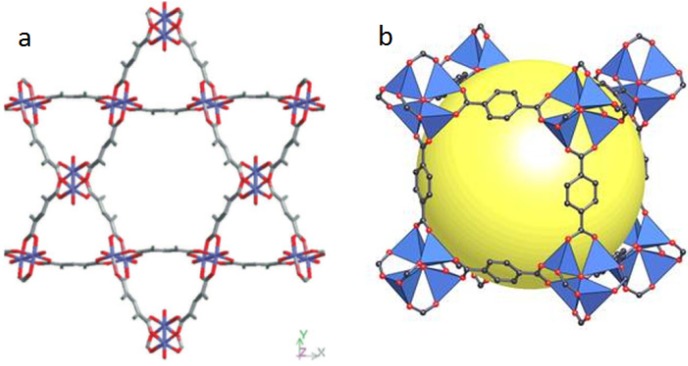
Remarkable MOFs: (**a**) HKUST-1, a dicopper(I) paddlewheel cluster structured by organic linkers, viewed along the cell body diagonal (111) (From [71]; reprinted with permission of AAAS) and (**b**) MOF-5, containing terephthalate anions structured by Zn_4_O clusters (reprinted from [72] with permission of Macmillan Publishers Ltd: Copyright 1999).

In 1959, Kinoshita *et al.* [66] reported on the first MOF, bis(adiponitrile)copper(I) nitrate, but it certainly did not fit the above-mentioned definitions. From the end of the 1950s until the beginning of the 1990s, only a few works on this theme were published and they did not encourage researchers to work in the field. Only in the first half of the 1990s did Robson and Yaghi revive studies on CPs with several notable papers [67,68,69,70]. Finally, in 1999, Chui *et al*. [71] and Li *et al.* [72] described two of the most cited MOFs, HKUST-1 and MOF-5, respectively. While the former is built from dicopper(II) tetracarboxylate paddlewheel clusters [Figure 3(a)], the latter bears terephthalate anions coordinated to Zn_4_O clusters [Figure 3(b)].

### 2.1. Strategies to Synthesize MOFs

The last decade saw advances in the field of MOFs. New synthetic approaches other than conventional heating systems were developed [73,74], such as microwave-assisted heating [75] and electro- [76], mechano- [77,78], and sonochemical [79] methods, using either conventional or high-throughput techniques [73]. More recently, Tanabe and Cohen [80] reviewed the progress in the post-synthetic modifications of MOFs, including the functionalization of building blocks in synthesized MOFs, a new strategy in solid-organic synthesis.

Currently, the synthesis of MOFs focuses not only on their building blocks, but also on their reticular structure [81]. To facilitate the identification of some kinds of nets within the metal-organic framework and to develop a standardized nomenclature for these materials, pioneer Yaghi and his group created the Reticular Chemistry Structure Resource (RCSR) [82,83], an open database for crystal nets containing names, symbols, examples, and keywords for crystal nets, polyhedra, and layered structures. This database can help systematize MOFs studies and classification. In 2009, three years after the publication of the RCSR database, Yaghi *et al.* defined Reticular Chemistry as “the chemistry of stitching molecular building units by strong bonds into extended structures” [83]. A new field now exists in chemistry that aims to understand the properties of MOFs.

Apart from RCSR, ligands, and nets, special attention has been paid to the clusters that constitute the MOF scaffold. These Secondary Building Units (SBU) consist of more than one metal cation interconnected by μ-oxo and carboxylate bridges, for example [84]. They can bind to the ligand groups from three to sixty-six points of extension, depending on: (i) the number of metal ions in the cluster, (ii) unsaturated coordination points, and (iii) metal atoms saturated by the ligands forming the cluster only [84].

Efficient purification methods to obtain MOFs are crucial when one thinks about applying these systems in catalysis [85]. Because MOFs are solid materials, they can be purified by density separation processes, where the MOF is suspended in an adequate solvent and solid compounds of different densities begin to separate in the dispersion. One way to open the internal channels of MOFs is to activate the structure under supercritical conditions. This process expels large solvent molecules and leaves the channels free for chemical reactions [85]. These two purification strategies are essential when using MOFs in catalysis, because the catalyst must be pure and accessible to substrates in the heterogeneous phase.

Not only does MOF purification deserve special care; indeed, a rational approach must be developed to control catenation, interpenetration, and other structuring processes that could render the material inappropriate for some applications [85]. The choice of linkers, concentration, and temperature can prevent catenation, providing MOFs for different purposes [85].

**Figure 4:**
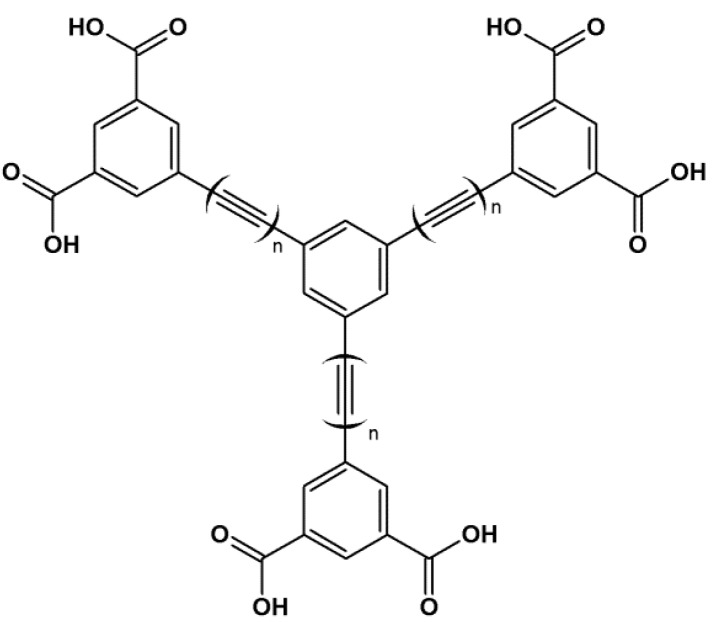
A 1,3,5-trisubstitued benzene with linear chains made of alternating single and triple bonds ending with a 3,5-dicarboxybenzene, a building block for MOF with ultra-high surface-areas [86].

High surface areas are also desirable when using MOFs, especially in catalysis. In 2012, Hupp *et al.* [86] prepared two MOFs with ultra-high surface areas; their materials overcame the range of 7,000 m^2^g^−1^, with Brunauer-Emmett-Teller (BET) surface areas of 7,010 and 7,140 m^2^g^−1^ and pore volumes of 3.75 and 4.40 cm^3^g^−1^ for NU-109E and NU-110E, respectively. In this same work, the authors conducted a theoretical study using a hypothetic MOF built from a 1,3,5-trisubstitued benzene with linear chains consisting of alternating single and triple bonds and ending with a 3,5-dicarboxybenzene (Figure 4) and one paddlewheel cluster for the carboxylate unit. The authors expected that this hypothetical material with long rigid carbon chains would present a BET surface area of up to 14,600 m^2^g^−1^.

## 3. MOFs with Porphyrins as Building Blocks

MOFs offer the features that are desirable in a catalyst, so catalysis is noteworthy among the numerous applications of MOFs in the fields of gas storage [87,88,89], separation processes [90], ion exchange [91], pre-concentrators [92,93], chemical sensors [94], drug delivery [95,96,97], and light harvesting [98]. In fact, the organic composition of the pores and walls of MOFs can be advantageous to catalysis. The hydrophobicity of the solid can be controlled by functionalization with linkers, which tailors the polarity of the MOF channels and pores and facilitates the approach of certain molecules to the catalyst. In a catalytic reaction, the MOF inorganic clusters can attract a polar reactant, while a nonpolar substrate can interact with the hydrophobic walls of the MOF and remain confined in the pores. This culminates in a local pre-concentration of the reactants and can help the reaction to occur.

Coordinative saturation of the metal ions is one of the challenges faced when someone tries to apply MOFs in heterogeneous catalysis. When all the metal centers have their coordination sites occupied by ligands (to form clusters or to support the structure), no sites are available to react with the substrate. Therefore, it is necessary to incorporate coordinatively unsaturated metal centers in the walls of the MOF. Using peripherally functionalized inorganic complexes as a building block for MOFs might be an alternative to circumvent the problem of coordinative saturation since they do not have a closed structure, involving the metal center and the metal may not be saturated by ligands. Schiff bases [99,100], bipyridines [101], ferrocenes [102,103], and MPs [64] are among the molecules that can act as metalloligands in MOFs.

With recognized catalytic activity in both homogeneous and heterogeneous phases, MPs can be the metalloligand of choice for MOFs exhibiting free redox sites on their walls. The rigid and planar 1-nm^2^ porphyrinic ring can be peripherally functionalized at the β-pyrrole or *meso* positions, as cited in Section 1.1 of this review. This functionalization can be achieved by using diverse Lewis-base groups, which are able to coordinate with other metal centers. 

**Figure 5:**
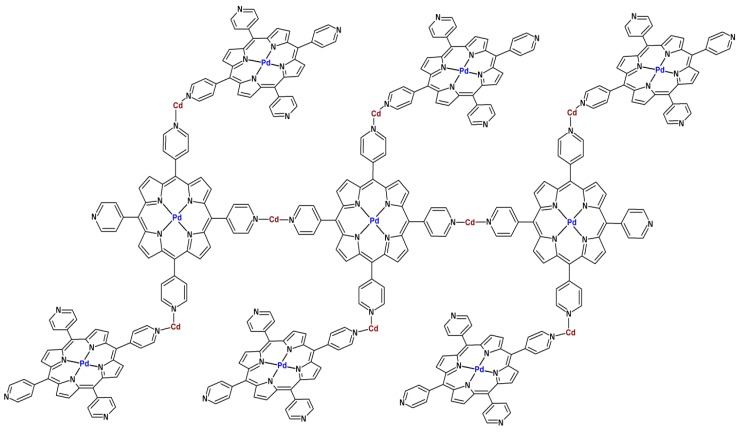
First porphyrinic MOF consisting of [tetra(4-pyridyl)porphyrinate] Pd(II) interconnected by cadmium(II) ions in a three-dimensional infinite net, as reported by Robson *et al.* [104]. Nitrato and aqua ligands have been omitted for clarity.

Robson *et al.* [104] described the first porphyrin-based MOF–[tetra(4-pyridyl)porphyrinate] Pd(II) connected by cadmium(II) ions, and not by SBUs (Figure 5). In this first porphyrinic MOF, the four pyridyl substituents of each porphyrin attach to a cadmium(II) ion. There are two kinds of connection in this material: one is the formation of linear porphyrinic chains, where each cadmium(II) ion is connected to two porphyrins in a *trans* fashion; the other connection involves the binding of the two cadmium ions of each porphyrin to another ring in *cis* mode. Two water molecules and nitrate ions complete the coordination sphere of the octahedral cadmium(II) ion. This arrangement confers a three-dimensional structure to the solid.

The “infinite polymeric network” depicted in Figure 5 was not only the first porphyrinic MOF, but also one of the reasons for the revival of the studies on inorganic-organic polymers in the 1990s. 

When clusters, SBUs, are not present in the structure of the MOF, the framework becomes fragile after gas and solvent removal. The PIZA family of porphyrinic MOFs represents the end of this problem: they contain clusters between the MP, affording more robust MOFs and making them applicable as catalysts [105]. Suslick *et al.* [105] were the first to report on the use of a porphyrinic MOF, PIZA-3 in catalysis. This material consists of a three-dimensional net of [tetra(4-carboxyphenyl)porphyrinate] Mn(III) connected by trinuclear manganese clusters. We will provide more details about the catalytic behavior of this MOF in Section 4.1.

Synthesis of porphyrin-based MOFs has increased in the last decade. At least four important families of these compounds exist: the pioneering *P*orphyrin *I*llinois *Z*eolite *A*nalogue (PIZA) [105], *P*orphyrin *P*illared *F*rameworks (PPF) [106,107], *R*obust *P*orphyrin *M*aterials (RPM) [108,109], and the most recent *M*etal-*M*etallo*p*orphyrin *F*rameworks (MMPF) [110,111,112,113,114,115]. These families are usually comprised of MOFs built from pyridyl and carboxyphenyl porphyrins and SBUs instead of single ions. These imino- and carboxy-substituted phenylporphyrins have Lewis basic sites that favor peripheral coordination with metal centers. The presence of SBUs in the structure furnishes a robust material that does not collapse after gas or solvent removal or even during the catalytic reaction [106].

## 4. Activity of Metalloporphyrin-Based MOFs in Heterogeneous Catalysis

The use of porphyrinic MOFs in heterogeneous catalysis is still in its early stages, but the scientific literature already brings some good examples that help minimize the problems inherent to homogeneous catalysis involving MP. Two kinds of catalyzed chemical reactions will be the scope of this mini-review: Lewis acid catalysis and oxidation reactions. These processes are widely employed worldwide and are usually conducted either in homogeneous phase or in heterogeneous media (with the chromophores immobilized onto a matrix).

### 4.1. Oxidation Reaction

PIZA-3, a microporous solid based on [tetra(4-carboxyphenyl)porphyrinate] Mn(III) connected by trinuclear manganese clusters, was the first porphyrinic MOF to be applied as catalyst [105]. In this MOF, each manganese porphyrin binds to four clusters, and each cluster attaches to eight porphyrins, giving rise to a three-dimensional material. The MP-based PIZA family of MOFs was the first class of porphyrinic MOFs to receive a special name, like *M*aterials of *I*nstitute *L*avoisier (MIL), *H*ong *K*ong *U*niversity of *S*cience and *T*echnology (HKUST), and *Po*hang University of *S*cience and *T*echnology (POST), among others [116].

PIZA materials are built from [tetra(4-carboxyphenyl)porphyrins] and different metals: PIZA-1 contains cobalt porphyrins linked by trinuclear linear clusters of Co(II); PIZA-2 has the same cobalt porphyrin as building block, but the MP are connected through trinuclear bent cobalt clusters; PIZA-3 is isostructural to PIZA-2, but its metal center is manganese; finally, PIZA-4 consists of zinc porphyrins coordinated by Zn_4_O clusters [105].

**Figure 6:**
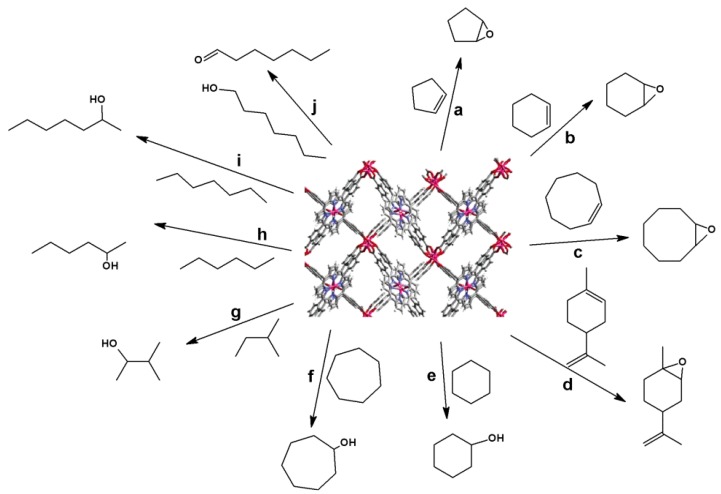
Oxidation reactions catalyzed by PIZA-3 (only desired products are shown). *a–d*: epoxidation of olefins; *e–f*: hydroxylation of cyclic alkanes; *g–i*: hydroxylation of aliphatic alkanes; *j*: oxidation of terminal alcohol to aldehyde. Reprinted (adapted) with permission from [105]. Copyright (2005) American Chemical Society.

The catalytic activity of PIZA-3 in the oxidation of hydrocarbons has been investigated (Figure 6), because manganese porphyrins are known to be efficient catalysts for oxidation reactions in both homogeneous [117,118,119] and heterogeneous phases [5,48]. Cobalt porphyrins also display catalytic activity toward oxidation reactions, but they are less reactive than the parent manganese complexes [120,121].

Reactions catalyzed by PIZA-3 have been accomplished using iodosylbenzene as the oxygen source and imidazole as cocatalyst, at a catalyst/oxidant/substrate/cocatalyst molar ratio of 1:10:1,000:1, in acetonitrile, at room temperature, for 2 h. This catalyst is robust: UV-vis spectroscopy detected no manganese porphyrin in the supernatant of the reaction, proving that the reaction is truly heterogeneous. The results achieved with PIZA-3 are similar to those obtained in homogeneous catalysis using manganese porphyrin and in heterogeneous reactions employing manganese porphyrins immobilized onto supports [105].

PIZA-3 selectively epoxidizes cyclopentene (23% epoxide), cyclohexene (23% epoxide), cyclooctene (74% epoxide), and limonene (20% epoxide) [Figure 6(a–d), respectively], with no allylic product being detected after the reaction. This is expected for all the substrates, except for cyclooctene [122]. The limonene branching is not epoxidized, which shows that the system selectively oxidizes the ring but not the terminal alkene of the substrate. The PIZA-3-catalyzed oxidation of alkanes is selective for the alcohol in the case of linear substrates with an alcohol/ketone ratio of approximately 8–8.9, achieved for the cyclic substrates cyclohexane (43% alcohol) and cycloheptane (47% alcohol) [Figure 6(e–f), respectively].

The hydroxylation of the linear alkanes hexane (17% total alcohols) and heptane (23% total alcohols) catalyzed by PIZA-3 gives similar yields for alcohols at positions 2 and 3 [Figure 6(h–i)]; *i.e.*, the catalytic reaction is not shape-selective. In this case, therefore, catalysis occurs on the external surface of the MOF, since the internal cavities would have selectively adsorbed small and hydrophilic molecules only. Secondary oxidation of 2-methylbutane was performed instead of oxidation of tertiary carbon, the labile position due to thermodynamic factors [123]. Hence, the solid PIZA-3 is active in all the reactions catalyzed by other manganese porphyrins, with the advantage that it is a heterogeneous catalyst that can be recovered at the end of the reaction and reused.

The RPM family of porphyrinic materials also displays catalytic activity in oxidation reactions. The material ZnMn-RPM was used to investigate the catalytic activity of RPMs. The structure of this solid is similar to that of ZnPO-MOF [124], except that a [tetra(4-carboxyphenyl)porphyrinate] Zn(II) and not a tetracarboxylic acid is used as spacer. The redox metalloligand is [(5,15-dipyridyl-10,20-bis(pentafluorophenyl))porphyrinate] Mn(III), the same porphyrin as the one in ZnPO-MOF [124], structured by paddlewheel dinuclear zinc(II) clusters [108]. This material acquires permanent microporosity after cycles of CO_2_ sorption; its surface area is approximately 1,000 m^2^g^−1^, a desired feature for heterogeneous catalysis. In the general nomenclature of the RPM family, the first metal of the acronym (M^1^) is related to the carboxyphenyl porphyrin, whereas the second metal (M^2^) corresponds to the central metal of dipyridyl porphyrin (M^1^M^2^-RPM).

**Figure 7:**
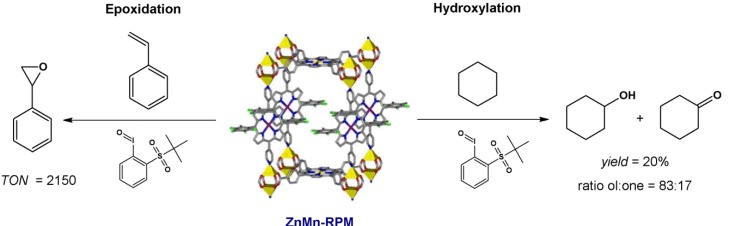
Epoxidation of styrene (left) and hydroxylation of cyclohexane (right) by an iodosylbenzene derivative, catalyzed by ZnMn-RPM. Reprinted (adapted) with permission from [108]. Copyright (2011) American Chemical Society.

Epoxidation of styrene catalyzed by ZnMn-RPM was carried out by using an oxidant analogous to iodosylbenzene – 2-(tert-butylsulfonyl)iodosylbenzene (TBS-PhIO, Figure 7). ZnMn-RPM performs well as a catalyst; it is more stable than [Mn(TPFPP)]Cl, [tetra(pentafluorophenyl)porphyrinate] Mn(III) chloride, a second-generation catalyst [8]. Whilst [Mn(TPFPP)]Cl affords a turnover number (TON) of 780 in homogeneous catalysis, due to total catalyst deactivation, ZnMn-RPM affords a TON of 2,150 after 800 min of reaction, after complete oxidant consumption [108]. An induction time is necessary for ZnMn-RPM though (about 250 min), because the reactants have to penetrate through the pores and channels of the MOF. This induction period can be reduced by using smaller, mechanically crushed particles. Catalysis is indeed heterogeneous: after removal of the MOF, the reaction solution does not display any catalytic activity; moreover, no manganese porphyrin leaches during the reaction.

Oxidation of cyclohexane catalyzed by ZnMn-RPM was also performed, using the same oxidant mentioned above. In this case, ZnMn-RPM furnished a total yield of 20%, with 83% and 17% selectivity for cyclohexanol and cyclohexanone, respectively. This result is lower than the one obtained in homogeneous catalysis using [Mn(TPFPP)]Cl and iodosylbenzene at a catalyst/oxidant/cyclohexane molar ratio of 1:10:1,000 (total product yield = 38%) [125], because it is difficult for the reactants to permeate the solid catalyst, as demonstrated by the induction time. 

In both cases (styrene epoxidation and cyclohexane oxidation), the MOF ZnMn-RPM exhibits excellent catalytic performance and offers advantages over homogeneous catalysis; for example, catalyst deactivation by bimolecular interaction (e.g., dimer formation) or self-destruction is prevented. Besides, MOFs enable catalyst reuse after their recovery from the reaction solution and rinsing. However, ZnMn-RPM maintains only two-thirds of its catalytic activity in a new reaction, because polymeric products from styrene block pores and channels in the solid catalyst [108].

**Figure 8:**
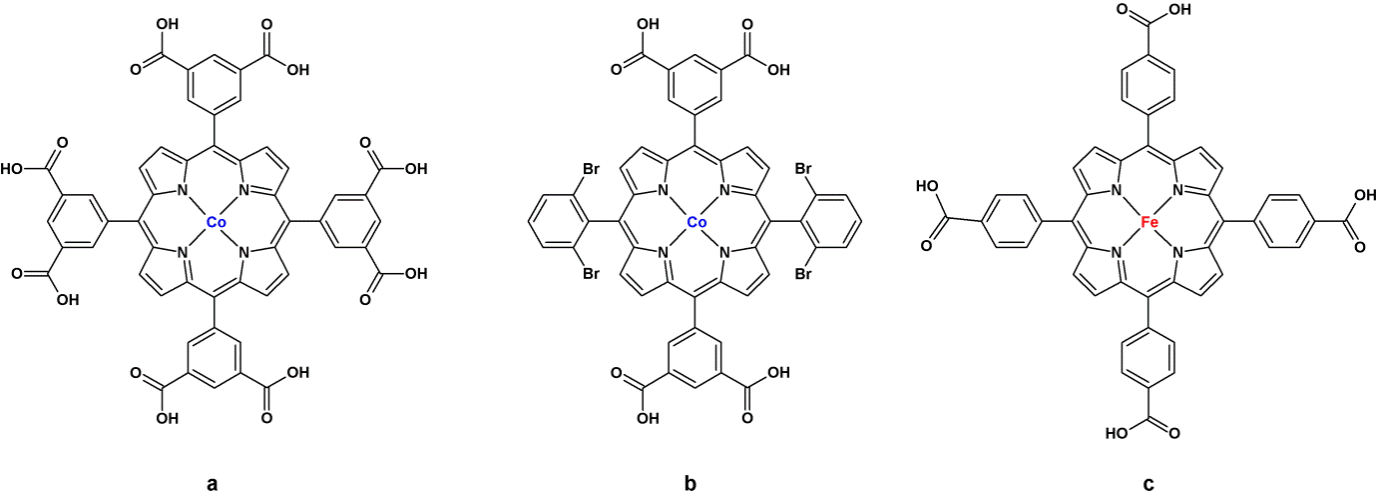
Building blocks of the catalytically active MMPF series. (**a**) *MMPF-2* and *MMPF-5*: [(tetra(3,5-dicarboxyphenyl)porphyrinate) Co(III)]; (**b**) *MMPF-3*: [5,15-bis(3,5-dicarboxyphenyl)-10,20-bis(2,6-dibromophenyl)porphyrinate) Co(III)]; (**c**) *MMPF-6*: [tetra(4-carboxyphenyl)porphyrinate] Fe(III)].

The recently described MMPF family of porphyrinic MOFs synthesized by Ma’s group since 2011 [110,111,112,113,114,115] consists of carboxyphenyl-substituted MP linked through SBUs with different metals (Cu, Co, Zn, Cd, Zr, Fe) and does not contain any additional linkers or spacers. Four members of the MMPF family, bearing redox metalloligands, have been shown to display activity in catalytic oxidation reactions: MMPF-2, MMPF-3, and MMPF-5(Co) efficiently catalyze the epoxidation of stilbene, whilst MMPF-6 presents peroxidase activity (Figure 8).

The first MMPF reported to be active in catalysis was MMPF-3, a three-dimensional material built from a solvothermal reaction between [5,15-bis(3,5-dicarboxyphenyl)-10,20-bis(2,6-dibromophenyl)-porphyrin] (DCPDBPP) [Figure 8(b)] and copper nitrate [113]. This porphyrin has four coordination points, two in each extremity of the porphyrin, forming an I(roman)-shaped linker; like the redox building blocks of the RPM family [108], the two other phenyl rings bear EWG, which enhance the catalytic activity of the MP in oxidation reactions [8]. The building blocks of MMPF-3, cobalt porphyrins, are structured by SBUs of dicobalt paddlewheel clusters; this furnishes a solid with **fcu** (face-centered cubic) topology, three kinds of polyhedral cages (cubohemioctahedron, truncated tetrahedron, and truncated octahedron), and a surface area up to 750 m^2^g^−1^ [Figure 8(b)] [113].

**Figure 9:**
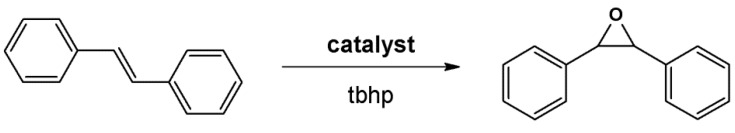
Epoxidation of *trans*-stilbene by a series of MOF and a cobalt porphyrin in homogeneous medium [113].

Meng *et al.* [113] applied MMPF-3 as catalyst in the epoxidation of *trans*-stilbene with *tert*-butyl hydroperoxide (tbhp) as oxidant (Figure 9), at a catalyst/oxidant/substrate molar ratio of 1:1,500:1,000 in acetonitrile, for 24 h. In these conditions, MMPF-3 affords conversion and epoxide yield of 95.7% and 87.1%, respectively. These results are better than those obtained from the following control reactions: (i) homogeneous catalysis using only the cobalt porphyrin [Co(DCPDBPP)], the building block of MMPF-3, as catalyst–conversion of 60.4% and selectivity of 67.0%; (ii) reaction employing another MOF with the same fcu topology as MMPF-3, namely fcu-MOF-1, which contains the tetracarboxylic acid H_4_bipa-tc as building block–conversion of 47.1% (nearly half that obtained for MMPF-3) and yield of 76.7%; and (iii) a blank reaction with no catalyst–conversion of 9.0% [113]. Together, these results show that cobalt porphyrins account for the major catalytic activity of MMPF-3; there is also some contribution from cobalt paddlewheel clusters, because fcu-MOF-1 is active, as seen from control 2.

In reference [113], the authors also compare MMPF-3 with two other previously synthesized metalloporphyrinic MOFs–MMPF-2 and PPF-1Co. Solid MMPF-2, obtained by Ma's group, bears [tetra(3,5-dicarboxyphenyl)porphyrinate] Co(II), an octatopic ligand, and trinuclear cobalt SBUs [Figure 8(a)] [111]. Choe *et al.* [126] reported on PPF-1Co, a MOF based upon [tetra(4-carboxyphenyl)porphyrinate] Co(II), a cruciform ligand, and SBUs consisting of dinuclear cobalt paddlewheel clusters, which provide a two-dimensional framework. Even though MMPF-2 has larger surface area, and despite the exposed cobalt sites in PPF-1Co, both solids yield lower conversion of *trans*-stilbene to the epoxide as compared with MMPF-3 (67.2% and 23.7% for MMPF-2 and PPF-1Co, respectively). Authors attribute these lower catalytic activities to the non-alignment of cobalt porphyrin sites with the center of channels or to their parallel orientation to channels, which reduces the number of sites available for catalysis.

Wang *et al.* [115] described another MMPF material that catalyzes the epoxidation of *trans*-stilbene: MMPF-5(Co), a MOF prepared via a post-synthetic method of linker exchange from MMPF-5, which in turn is a cadmium(II)-based MOF with [tetra(3,5-dicarboxyphenyl) porphyrinate] Cd(II) connected by cadmium(II) clusters. In this method, all the cadmium porphyrins are exchanged for cobalt porphyrins [Figure 8(a)], but structural cadmium(II) remains intact, so MMPF-5(Co) retains the same crystalline nature as MMPF-5. MMPF-5(Co) catalyzes the oxidation of the alkene to the epoxide with 87.0% conversion *versus* 28.1% obtained using homogeneous catalysis with a similar cobalt porphyrin, [tetra(3,5-dicarboxymethylesterphenyl) porphyrinate] Co(II). These results attest that MOF is resistant to catalyst deactivation. Control tests with MMPF-5 and without any catalyst provide a conversion of only 9%, proving that cadmium units do not have activity in this catalytic reaction [115].

**Figure 10:**
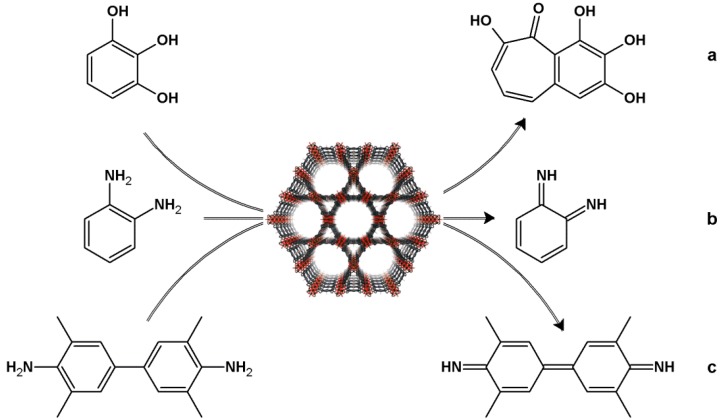
PCN-222(Fe)-catalyzed oxidations of (**a**) THB to purpurogallin; (**b**) OPD to *o*-quinonediimine; and (**c**) TMB to the respective diimine (adapted from [127] with permission. Copyright © 2012 WILEY-VCH Verlag GmbH & Co. KGaA, Weinheim).

Zhou *et al.* [127] were the first to report the synthesis of PCN-222(Fe) (PCN = Porous Coordination Network), a material based on [tetra(4-carboxyphenyl)porphyrinate] Fe(III) as building block [Figure 8(c)] and structured by hexanuclear zirconium clusters. PCN-222(Fe) consists of a three-dimensional porous material (pore diameter equal to 1.56 cm^3^g^−1^) with high surface area (2,200 m^2^g^−1^). The authors applied this MOF to catalyze the oxidation of three different diagnostic substrates for heme-like mimetic activity: 1,2,3-trihydroxybenzene [THB, Figure 10(a)], *o*-phenylenediamine [OPD, Figure 10(b)], and 3,3,5,5-tetramethylbenzidine [TMB, Figure 10(c)]; they used hydrogen peroxide at a constant concentration as oxidant and varied the concentration of the substrate, to evaluate kinetic parameters.

The catalytic activity of PCN-222(Fe) was compared with the activity of two biological systems, protoporphyrin IX (hemin) and horseradish peroxidase (HRP). HRP displays the best catalytic activity (higher values of *k*_cat_) and has the most affinity for substrates (higher values for *K*_m_), as expected for a natural enzyme; hemin furnishes the worst results regarding the oxidation of the substrates THB, OPD, and TMB. In turn, the MOF PCN-222(Fe) exhibits excellent catalytic activity, with *k*_cat_/*K*_m_ ratios only two orders of magnitude lower than the same ratios obtained with HRP. This MOF performs better in THB oxidation and requires only a small concentration of the substrate; HRP is better for OPD oxidation. PCN-222(Fe) is selective for smaller substrates, because it has smaller channels. This is an advantage of PCN-222(Fe) over HRP: the former has a density of active sites that is about 34 times greater than the density verified for HRP [127].

The last MMPF solid described to possess catalytic activity is MMPF-6, which is isostructural with PCN-222(Fe) [127], since they both bear the same building blocks and SBUs [114]. The three-dimensional MMPF, with an iron porphyrin loading of 6.18 × 10^−4^ g mol^−1^, also displays activity comparable to heme in two different reactions: oxygen and electron transfer in HEPES buffer, a biological-like environment. 

**Figure 11:**
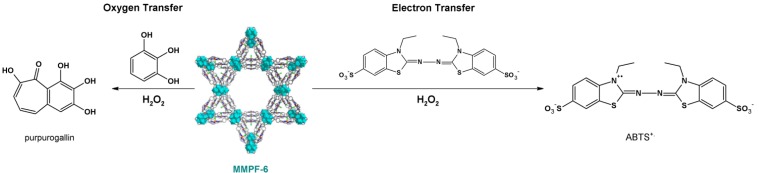
Oxidations catalyzed by MMPF-6 (middle): oxygen transfer to 1,2,3-trihydroxybenzene, to form purpurogallin (left), and electron transfer to ABTS, to give the pi-cation ABTS^+·^ (right). Reprinted (adapted) with permission from [114]. Copyright (2012) American Chemical Society.

Oxygen transfer from hydrogen peroxide to THB, which gives purpurogallin (Figure 11, left), has an initial rate equal to one-third of the rate observed for free myoglobin (Mb) in the same buffer. Electron transfer involving ABTS, to generate π-cation ABTS^+·^ (Figure 11, right), is about one-fourth as fast as the rate achieved with free Mb in solution. MMPF-6 is also active in ethanol, a solvent where enzymes generally do not have catalytic activity. In this case, oxygen and electron transfer are enhanced 107 and ~215 times as compared with the blank reaction (no catalyst), respectively. Zirconium oxide clusters are not involved in the catalytic cycles, as demonstrated by the use of free-base porphyrin as the building block instead of the iron(III) complex [114].

Yang *et al.* [128] have employed three isostructural porphyrinic MOFs from the ZJU family (ZJU = Zheijang University), based on [tetra(3,5-dicarboxyphenyl)porphyrin] and di- and trinuclear metal clusters, as catalysts for the oxidation of alkylbenzenes. Each carboxyl substituent from the phenyl ring of these MOFs is linked to a di- and a trinuclear manganese cluster, in a symmetric design, to afford a net with tbo (twisted boracite) topology, the same topology that was found for HKUST-1 [71]. ZJU-18 is built from manganese porphyrin and manganese clusters, ZJU-19 has nickel(II) porphyrin as building block and manganese SUBs, and ZJU-20 consists of manganese porphyrins connected by cadmium clusters [128]. In the oxidation of ethylbenzene to acetophenone by TBHP, homometallic ZJU-18, the nickel porphyrin-based material ZJU-19, and the cadmium-SBU-based ZJU-20 yields up to 99, 9%, and 69% conversion to ketone, respectively. Homogeneous catalysis with manganese(III) and nickel(II) porphyrins affords conversions to ketone of 16% and only traces, respectively. Together, these results show that the building block manganese porphyrin is the catalytically active species in the ZJU family, and that the Mn-SBU clusters contribute to oxidative reactions. ZJU-18 is better than the catalyst in homogeneous phase, because the MOF structure avoids self-oxidation and dimerization of the MP.

The good performance of ZJU-18 prompted researchers to test its activity in the oxidation of longer-chain alkylbenzenes. The larger the alkyl chain, the worse the catalytic performance of this metalloporphyrinic MOF, suggesting that catalysis depends on the channels of the material. Oxidation of diphenylmethane, for which ZJU-18 gives lower conversion as compared with homogeneous medium (18% and 26% conversion, respectively), confirms this assumption. This pattern is also true for the catalytic oxidation of 4-phenyl-ethylbenzene by ZJU-18 (conversion of 16% and 28% in heterogeneous phase and homogeneous medium, respectively), but not for smaller substrates. All these observations corroborate the idea that catalysis occurs only on the external surface of the MOF for larger substrates, whilst it takes place inside the pores in the case of smaller hydrocarbons. Therefore, ZJU-18 is size- and shape-selective [128].

Lee *et al.* [129] have employed another manganese porphyrin-based MOF in oxidation reactions: the building block is a porphyrin bearing two carboxylic terminations on opposite sides of the ring, forming a linear linker; the other two mesophenyl rings contain diethoxy groups at the *ortho* positions, but no strong EWG exists in the structure. Indium(III) is the structural metal, because its strong binding to carboxyl groups furnishes a more stable material. Uniform hexagonal rods with a small surface area of 77.7 m^2^g^−1^ originate after reaction between the manganese porphyrin and In(NO_3_)_3_, due to collapse of the framework. This MOF catalyzes the epoxidation of styrene by TBS-PhIO at a catalyst/oxidant/substrate molar ratio of 1:1,000:2,000, in chloroform. Comparing the MOF with its building block molecule, [5,15-bis(4-carboxyethylphenyl)-10,20-bis(2,6-diethoxyphenyl)-porphyrinate]Mn(III), the lifetime of the catalyst triples on going from homogenous to heterogeneous catalysis. The MOF affords a TON in the range of 900, and it can be reused for at least five times while elevated TONs are maintained. Catalysis is indeed heterogeneous, because no MP leaches from the MOF during the reaction [129].

Xie *et al.* [130] have also performed the oxidation of styrene using a MOF based on [tetra(carboxyphenyl)porphyrinate]Pd(II), [Pd(TCPP)]. In this material, each [Pd(TCPP)] metalloligand binds to eight dinuclear cadmium centers, to form a three-dimensional framework displaying distorted channels. The solid is still robust and resistant after solvent removal, and retains its crystalline structure. In the presence of acids, palladium catalysts are selective toward acetophenone instead of epoxide, as seen in the examples cited for other metals in this same section. In the absence of acids, the oxidation of styrene by H_2_O_2_ in the presence of this MOF does not occur. Addition of perchloric acid to the reaction medium leads to 100% conversion, with 91% and 9% selectivity for acetophenone and benzaldehyde, respectively. This MOF furnishes better results than those obtained with [Pd(TCPP)] in homogeneous medium (90% conversion and 78% selectivity for acetophenone). In addition, strong oxyacids (i.e., HClO_4_, H_2_SO_4_, HNO_3_) promote higher conversion than a hydracid (HCl) or an organic acid (HOAc). The system is heterogeneous in nature and the catalyst can be recovered and reused in other cycles [130].

More recently, Wu *et al.* [131] have synthesized and tested four different metalloporphyrinic frameworks as catalysts for oxidation and aldol reactions [131]. These MOFs are based on [M(III)(TCPP)] (M = Mn and Fe) bearing paddlewheel clusters of cadmium(II) or zinc(II) as SBUs. The manganese materials are isostrucutural three-dimensional frameworks built from two-dimensional nets of manganese porphyrins connected by cadmium(II) or zinc(II) paddlewheel clusters; these clusters are pillared by formate bridges established between the SBUs and the manganese centers of the MP. The manganese(III) ion is five-coordinate and contains a formate unit as axial ligand, in an AB fashion [106,107,131]. The MOF having iron porphyrin as building block and zinc(II) paddlewheel clusters as SBUs also involves pillaring of two-dimensional nets of MP; the difference is that the SBUs connect via carboxylate bridges, in an AA model, and there is no axial ligand in the iron site [106,107,131]. When cadmium(II) is used as a structural metal with the iron porphyrin, the latter acts as a pentatopic ligand–it links to five cadmium(II) ions via three carboxylate units established with the other protonated carboxylates. The three-dimensional structure originates from dimerization of the iron centers in a low-symmetry network of superposed lamellae [131]. The catalytic activity of these MOFs has been tested in the epoxidation of a series of olefins by iodosylbenzene; more specifically, styrene, cyclopentene, cyclohexene, cyclooctene, terminal linear alkenes (1-hexene, 1-octene, and 1-dodecene), stilbene, and some modified stilbene and styrene substrates. The iron porphyrin-based MOF does not catalyze any of the epoxidation reactions: it undergoes self-destruction, as evidenced by bleaching of the reaction dispersion [131]. The manganese-based solid furnishes 100% product yield during styrene epoxidation, even though one of the axial positions of the MP is blocked by a bridge established with the SBU. The solid consisting of manganese porphyrin and zinc(II) paddlewheel clusters is also active for the epoxidation of other olefins, with yields close to 100% in some cases. Catalysis is really heterogeneous and happens on the external surface of MOF; the pores present in the solid do not allow the approach of the reactants to the manganese sites inside the network.

Because the iron porphyrin-based MOF is unstable in oxidation reactions, it has been applied as catalyst in the aldol reaction between nitrobenzaldehydes and methyl ethyl ketone (MEK). This MOF leads to good results in these coupling reactions, attributed to the high density of Lewis acids in the material.

### 4.2. Lewis Acid Catalysis

Hupp *et al.* [124] have synthesized the only reported example of a porphyrinic MOF that is able to catalyze acyl transfer reaction–ZnPO-MOF, obtained by solvothermal reaction between zinc(II) nitrate, [5,15-dipyridyl-10,20-bis(pentafluorophenyl))porphyrin], and [1,2,4,5-tetrakis(4-carboxy-phenyl)benzene] (H4TCPB), an octatopic oxygenated ligand, in DMF at 100 °C for five days. This MOF consists of four zinc porphyrins bearing the pyridyl groups in the vertical position, attached to dinuclear paddlewheel zinc clusters, which in turn bind to an octatopic linker that acts as spacer. The use of organic molecules as spacers combined with metalloligands can provide larger channels and pores than those obtained using metalloligands only [106].

Octatopic spacers have been applied to obtain the RPM family of porphyrinic MOFs [108,109]. These isostrucutral materials have been developed by employing the same porphyrin or [tetra(carboxyphenyl)porphyrin], the spacer, and the metals Al, Zn, Fe, and Mn. ZnPO-MOF can be considered a member of the RPM family, although it was synthesized before this family was defined. As established by PXRD and TG, ZnPO-MOF is robust; it is also porous, with a surface area of approximately 500 m^2^g^−1^, as determined by the BET method (supercritical CO_2_ adsorption). All the zinc porphyrin sites in ZnPO-MOF are accessible to substrates [124]. 

**Figure 12:**
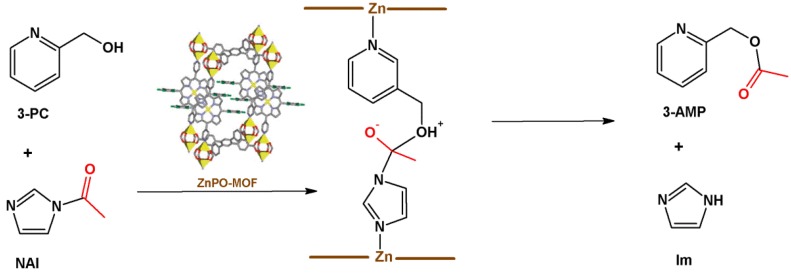
Acyl transfer from N-acetylimidazol (NAI) to 3-pyridylcarbinol catalyzed by ZnPO-MOF. In the MOF structure, zinc atoms are represented in yellow, oxygen in red, nitrogen in blue, fluorine in green, and carbon in grey. Reprinted (adapted) with permission from [124]. Copyright (2009) American Chemical Society.

Acetylation of 3-pyridylcarbinol (3-PC) using *N*-acetylimidazole (NAI) in acetonitrile, at 60 °C, in the presence of ZnPO-MOF was carried out by Hupp *et al.* [124]; the products were 3-acetoxy-methylpyridyne (3-AMP, the acetylated product) and imidazole (Im) (Figure 12). Zinc-containing supramolecular systems usually catalyze this reaction [132], which depends on the cavity that the co-facial zinc sites form. Acyl transfer reaction in the presence of ZnPO-MOF is 2,420-fold greater as compared with the uncatalyzed reaction. The reaction rate is higher than that obtained in the presence of another MOF containing only organic linkers in the structure–Zn_2_(*p*-terephthalate)_2_(4,4’-bipy). This result shows that the catalytic activity depends on the metalloligand and not on the paddlewheel zinc clusters. To investigate the importance of the self-assembled MP in the catalysis, the authors carried out the reaction in the presence of a solution of monomeric [tetra(phenyl)porphyrinate] Zn(II), [Zn(TPP)], in homogeneous medium. This system produced 3-AMP at lower yields than the systems based on the MOFs, attesting to the importance of having a catalyst involving the zinc porphyrin in a solid structure [124]. The absence of EWG at the phenyl rings also explains the lower yield achieved with homogeneous [Zn(TPP)]. In ZnPO-MOF, fluorine atoms are EWG and withdraw the electron density from zinc(II), making the metal center more electrophilic and favoring the coordination of cyclic imines. 

Hupp *et al.* [124] have envisioned three hypotheses to explain this mechanism:

(i) *Lewis acid activation*: preliminary coordination of NAI to a zinc-porphyrin site withdraws the electron density from the carbonyl group, generating the negative charge (Figure 12, center).
(ii) *Concentration of the substrates*: ZnPO-MOF cavities can concentrate the substrates and increase the reaction rate without changing the activation energy.
(iii) *Preferred orientation of reactants*: ZnPO-MOF cavity size (Zn-Zn distance = 11.6 Å) allows a pair of 3-PC and NAI to align, providing the ideal orientation for the reaction to take place.

To determine which mechanism acts during acetylation, Hupp *et al.* [124] conducted a study using isomers of 3-PC, namely 2-PC and 4-PC, to evaluate the dependence of the system on orientation [mechanism (iii)]. While 2-PC cannot coordinate to a zinc site due to the steric hindrance posed by the alcoholic group vicinal to nitrogen atom, reaction with 4-PC occurs with no significant change in the reaction rate as compared with 3-PC. This indicates that the catalytic system does not depend on a certain alignment of reactants, dismissing mechanism (iii)*.* As already discussed, using [Zn(TPP)] as catalyst gives much lower product yields as compared with ZnPO-MOF. Therefore, Lewis acid activation does not contribute to the catalytic in a majority stake. Adding 10% imidazole to the reaction mixture considerably reduces the reaction rate, showing that the product acts as inhibitor. All these findings considered, the authors proposed that the reaction takes place through a combination of mechanisms (i) and (ii), including a local pre-concentration of the reactants as well as a low Lewis acid activation factor [124].

Three years later, Roy *et al.* [133] published a paper on the computational calculations of some parameters of the reaction discussed above. In an elegant way, in this paper they re-propose the mechanisms of acyl transfer reactions. To begin with, these authors focus on the pre-concentration of reactants to develop a tool that calculates the rate constants of the most important steps involved in the mechanism. First, they determine the binding characteristics of reactants, products, and solvent molecules to zinc catalytic sites by DFT. Next, on the basis of these data, they calculate the equilibrium constants of molecules binding to and dissociating from ZnPO-MOF that depend on temperature. Finally, they come up with a kinetic model for heterogeneous catalysis, to determine which pre-concentrated components enhance the reaction rate. This methodology can be applied not only to the ZnPO-MOF system, but also to all the heterogeneous systems involving a pre-concentration step as the determinant of a catalyzed chemical reaction.

Roy *et al.* [133] also proposed equilibrium constants by considering that all the zinc porphyrin sites in ZnPO-MOF bind to solvent molecules (acetonitrile) before the reactants are added. In this way, the major steps involved in the reaction are the dissociation of the bond between catalyst and solvent (MOF-S), followed by binding of the reactant to the catalyst (MOF-R) [Equation (1)]. After the reaction, the second process consists of dissociation of the products from the catalyst (MOF-P) and rebinding of solvent molecules (MOF-S), which ends the reaction cycle [Equation (2)]:

MOF-S + R → MOF-R + S
(1)

MOF-P + S → MOF-S + P
(2)

The authors thus built the kinetic model on the basis of these two reactions and took into account the stoichiometric reaction for both homogeneous and heterogeneous catalysis. Because the zinc(II) cations of the paddlewheel clusters are pentacoordinated, they do not act as active sites for catalytic reactions; therefore, only porphyrinic zinc functions as catalyst.

The paper published by Oliveri *et al.* [132] confirmed many of the hypotheses put forward by Hupp *et al.* [124]. Both reactants–the substituted imidazole and pyridine–coordinate to the zinc sites via the nitrogen atom, because the energy of the Zn-N bond is greater than the energy of the Zn-O bond, facilitating the reaction.

Thermodynamic parameters can also explain why 2-PC does not react with NAI but 3-PC and 4-PC do. The binding energy between 2-PC and ZnPO-MOF is 8.6 kcal mol^−1^, whereas this same energy is 14.1 kcal mol^−1^ between 3-PC or 4-PC and ZnPO-MOF. The alcoholic substituent next to the nitrogen in 2-PC probably hinders the coordination between 2-PC and ZnPO-MOF, so the reaction fails. All the binding energies between reactants and catalyst are greater for ZnPO-MOF as compared with [Zn(TPP)] and help to explain why a better catalytic activity is achieved in the heterogeneous phase. Hence, at high concentration of products, this strong binding can inactivate the zinc sites. This situation can be reverted by modifying the temperature. Indeed, the dissociation constant between the products and MOF rises with increasing temperature. In this way, pre-concentration governs the acyl transfer reaction due to a high local concentration of substrates inside the cavities of ZnPO-MOF. 

Roy *et al.* still offer some directions to improve the reaction yield: reducing the temperature in the beginning of the reaction favors the pre-concentration of reactants; gradually increasing it eliminates product molecules coordinated to ZnPO-MOF [133].

The RPM MOF ZnAl-RPM catalyzes the ring opening of epoxides [109]. Hupp *et al.* [109] prepared this material from ZnZn-MOF, the only homometallic MOF of this family, using a post-synthetic method. Replacing [(dipyridyl)porphyrinate]Zn(II) with aluminum(III) dipyridyl porphyrin yields ZnAl-RPM, which is isostructural to the starting material and to all the other RPM materials, such as the ZnMn-RPM described in Section 4.1.

**Figure 13:**
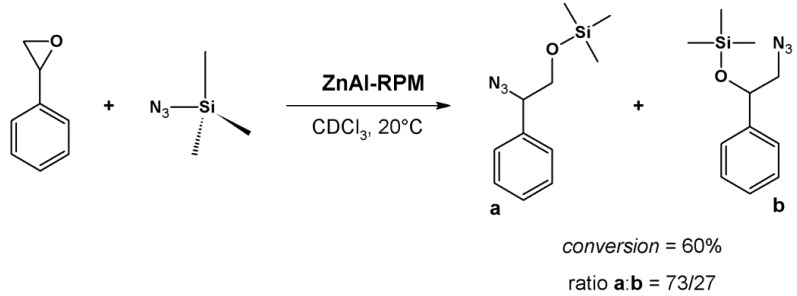
Ring opening of styrene oxide catalyzed by ZnAl-RPM.

Hupp *et al.* [109] investigated the ring opening of styrene oxide by trimethylsilylazide (TMSN_3_) catalyzed by ZnAl-RPM and other MOFs of this family (M^2^ = Zn(II), 2H^+^, Sn(IV), and Co(III)). The conversion of styrene to the azide is 60%, with selectivity for the less hindered product (Figure 13). Other RPMs bearing free base, zinc, cobalt, or tin dipyridyl porphyrin do not catalyze epoxide ring opening (conversion between zero and 6%); only aluminum can accomplish adequate opening, because this reaction depends on strong acid catalysis. The presence of axial ligands in the tin porphyrin can inactivate sites that catalyze the reaction, accounting for the low conversion [109]. Results with free base and zinc porphyrin dismisses the hypothesis that Zn_2_ paddlewheel clusters are catalytically active during the reaction; indeed, the core of active sites lies on [(dipyridylbispentafluorophenyl)porphyrin] only.

## 5. Conclusions

Metalloporphyrins are versatile molecules with different functions in biological systems. Moreover, they act as efficient and selective catalysts in various chemical reactions, which has inspired various research groups in the area of catalysis to evaluate the activity of these complexes in a number of catalytic systems. 

Researchers started by investigating the use of metalloporphyrins as catalysts in oxidation reactions conducted in homogeneous media. Significant results led them to synthesize more robust structures that resisted the oxidizing conditions of the catalytic reactions. Attention was also devoted to the proper choice of metal and oxygen donor. 

Immobilization of these complexes onto rigid, inert, and robust support solids paved the way for heterogeneous processes with technological applications—it was possible to recycle and reuse the developed catalytic solids, allowing for their application in larger scale. 

A newer strategy was adopted with a view to using this valuable and versatile molecule in economically viable heterogeneous catalytic processes in the future: preparing new materials based on the catalytically active molecule only, such as coordination polymers (CP) and metal-organic frameworks (MOFs). These solids are good candidates as catalysts of heterogeneous processes as opposed to the materials based on dilution and immobilization of the catalyst.

In this mini-review, we have provided a brief bibliography of the efforts recently dedicated to the development of MOFs bearing metalloporphyrins as building blocks. We have reviewed some synthetic approaches and the application of MOFs in catalysis. 

In the light of the manuscripts reviewed here, it is clear that the last two decades have seen many efforts toward the preparation and characterization (hard phase) of solids of this interesting family of compounds as well as the use of MOFs in productive, efficient, and recyclable catalytic processes.

This field still has many challenges to overcome, such as reusability through the maintenance of the MOF structure after a number of catalytic cycles; to keep the robustness of the material, under different reaction conditions; to access new selectivities just changing the metal source, peripheral porphyrin substituents and/or the topology of the material and to be active under soft conditions with good catalytic performance, arising the principles of green chemistry. So many kinds of chemical reactions catalyzed by metalloporphyrins in homogenous phase were not still investigated in heterogeneous medium with MOFs based on these chromophores (*i.e*., carbene insertion, cyclopropanation, reduction and DNA cleavage) and are the new challenges of this field. As Champness wrote [81], “MOFs are here to stay”, and its chemistry is still growing, so these porphyrinic materials still have long different ways to run with numerous new possibilities. 
